# Sleep Disturbance Mediates the Associations Between HIV Stigma and Mental and Physical Health Among Black Adults with HIV

**DOI:** 10.1007/s40615-024-02083-0

**Published:** 2024-07-11

**Authors:** Lu Dong, Laura M. Bogart, Matt G. Mutchler, David J. Klein, Madhumita (Bonnie) Ghosh-Dastidar, Sean J. Lawrence, Kathy Goggin, Glenn J. Wagner

**Affiliations:** 1 RAND Corporation, 1776 Main Street, Santa Monica, CA USA; 2 APLA Health, Los Angeles, CA USA; 3 Children’s Mercy Kansas City and University of Missouri-Kansas City Schools of Medicine and Pharmacy, Kansas City, MO USA; 4 Department of Psychiatry, Charles R. Drew University of Medicine and Science, Los Angeles, CA USA; 5 School of Public Health and Health Sciences, California State University, Dominguez Hills, Carson, CA USA; 6 Department of Psychology, San Diego State University, San Diego, CA USA

**Keywords:** HIV stigma, Mental health, Physical health, Sleep, Black Americans

## Abstract

**Objectives:**

Black Americans have been disproportionally affected by the HIV epidemic, and experience significant disparities in sleep health, mental health, and physical health domains. Using longitudinal data from a sample of Black adults with HIV, the current study examined the associations between stigma and mental and physical health outcomes and how sleep disturbance may play a mediating role.

**Methods:**

Data were drawn from a recent randomized controlled trial. Questionnaires were used to examine internalized and anticipated HIV stigma, perceived discrimination (enacted stigma) based on multiple social identities (i.e., HIV-serostatus, race, sexual orientation), sleep disturbance, mental health problems (depressive and posttraumatic stress disorder [PTSD] symptoms), and mental and physical health-related quality of life (HRQOL) at baseline, 7-month follow-up, and 13-month follow-up assessments. Linear mixed modeling was used to examine main effects of stigma on health outcomes; causal mediation analysis was used to estimate indirect paths through sleep disturbance.

**Results:**

Internalized and anticipated HIV stigma and multiple discrimination were associated with more sleep disturbance, more depressive and PTSD symptoms, and poorer mental and physical HRQOL. Results also indicated significant indirect paths (i.e., mediation) through greater sleep disturbance between HIV-related stigma and discrimination and mental health and health-related quality of life.

**Conclusions:**

Results support that sleep disturbance is a mediating pathway through which different forms of stigmas impact health outcomes. Sleep may be an intervention target to help improve mental and physical well-being and reduce health disparities among racial and ethnic minority people with HIV.

**Supplementary Information:**

The online version contains supplementary material available at 10.1007/s40615-024-02083-0.

## Introduction

Black Americans are overburdened among people with HIV in the United States. While representing about 12% of the U.S. population, Black individuals accounted for a disproportionately higher number of new HIV diagnoses (42%), HIV prevalence (43%), and HIV deaths (43%) than any other racial/ethnic groups in the U.S. in 2020 [1]. Black women and sexual minority men have been particularly hard hit by the HIV epidemic [2]. For example, Black women over 13 years-old are 11 times more likely than non-Hispanic White women to be diagnosed with HIV; Black sexual minority men have the highest percentage of new HIV diagnoses among sexual minority men across all races/ethnicities [2]. In addition, mental health problems such as depression and posttraumatic stress disorder (PTSD) are significantly more prevalent among people with HIV than among the general population [3]. Evidence shows that mental health problems disproportionally affect Black women living with HIV [4–6] and Black sexual minority men [7], compared to other subpopulations with HIV.

Internalized, anticipated, and enacted stigma from HIV and related marginalized identities (e.g., Black race, sexual minority orientation) are contributing factors to the mental and physical well-being of people with HIV [8–10] and specifically in Black Americans living with HIV [11–13]. Internalized HIV stigma refers to a person’s acceptance and adoption of negative societal beliefs and feelings and the social devaluation associated with HIV. Internalized stigma, including HIV-related stigma, is associated with poor mental health, including greater depressive symptoms, PTSD symptoms, and psychological distress, as well as lower psychological well-being and health-related quality of life [9, 10, 14–18]. Anticipated HIV stigma refers to expectations of discrimination or bias by others that are associated with being HIV-positive. Enacted HIV stigma refers to the experiences or perceived discrimination related to being HIV positive. Both anticipated and enacted stigmas involve experiences with others and, when occurring over time, are considered chronic stressors that can lead to poor mental and health outcomes [19].

Sleep disturbance is one of the most prevalent but underrecognized problems affecting people with HIV [20, 21], with up to 70% of people with HIV experiencing sleep problems [22–25]. Sleep disparities have been documented for Black Americans [26], including those living with HIV [27]. For example, Black men living with HIV reported poorer self-reported sleep quality and showed greater polysomnography-assessed sleep disturbance than seronegative controls [27]. While numerous structural-level factors (e.g., built environment such as neighborhood characteristics) contribute to sleep disparities [28, 29], research also shows that individual-level factors, including health-related stigma and perceived discrimination, also contribute to poor sleep in marginalized and minority populations [30, 31]. For example, a recent systematic review applied the Health Stigma and Discrimination Framework to examine sleep outcomes and found consistent cross-sectional associations between different health-related stigmas and self-reported sleep problems [30]. The Health Stigma and Discrimination Framework postulates how health-related stigmas manifest as stigma experiences (e.g., internalized and anticipated stigma, enacted stigma/discrimination) to influence a broad range of health-related outcomes [32]. However, as evident from this review, the associations between health-related stigmas and sleep have rarely been examined using longitudinal data in racial and ethnic minority individuals with HIV or incorporated intersecting stigmas and experiences based on other social identities such as race and sexual orientation. The current study aimed to address these gaps.

Sleep is a critical process that supports the maintenance of mental and physical health and has been identified as a transdiagnostic process common to a range of mental disorders and physical health domains [33, 34]. Disrupted sleep is a symptom or clinical characteristic across virtually all mental disorders, including depression and PTSD. Sleep disturbance has increasingly been recognized as a contributory causal factor in the onset and maintenance of major mental disorders. Although sleep disturbance and mental disorders are often bidirectionally associated, the strongest causal pathway is typically from sleep disturbance to mental health symptoms [35]. Sleep also mechanistically contributes to physical health functioning, including growth and stress hormones, immune systems, and cardiovascular health [29, 33, 36]. As such, a key aspect of the current study examines sleep as an indirect path or mediating factor through which stigma and discrimination experiences negatively impact mental and physical health outcomes.

Sleep is particularly sensitive to the social and physical environment [37]. Experiences of stigma and discrimination can contribute to allostatic load and induce acute and chronic stress, manifested as sympathetic nervous system activation and cortisol release, which in turn negatively affects sleep quality and quantity at night [37, 38]. Some cross-sectional studies have supported that greater perceived stress and increased anxiety may partially explain the link between discrimination (e.g., experiences of unfair treatment) and poor sleep outcomes in racial and ethnic minority individuals [39, 40]. Given the essential role of sleep in supporting mental and physical health [33], it is plausible that sleep may be an important mediating path linking the experiences of stigma and discrimination to poor mental and physical health outcomes. Examining these links is particularly important for groups that experience significant health disparities, given that poor sleep may be an understudied factor contributing to health disparities [29].

Using longitudinal data from a randomized controlled trial (RCT), the current study examined the relationships among stigma/discrimination, sleep, and mental and physical health outcomes assessed at baseline, 7-month follow-up, and 13-month follow-up in a sample of Black men and women with HIV and tested sleep disturbance as a potential mediator or indirect path, while controlling for age, sex assigned at birth, and intervention condition. Specifically, we examined whether internalized and anticipated HIV stigma and multiple discrimination (on the basis of HIV status, Black race, and gay sexual orientation) were associated with sleep, mental health, and physical health outcomes across three time points (baseline, 7-month follow-up, and 13-month follow-up). We hypothesized that higher levels of HIV-related stigma and discrimination would be predictive of more sleep disturbance, poorer mental health outcomes (i.e., greater depressive symptoms, more PTSD symptoms), and worse quality of life in both mental and physical health. We then examined whether sleep problems served as an indirect path between stigma/discrimination and mental and physical health outcomes. We hypothesized that greater experiences of HIV-related stigma and multiple discrimination would be associated with mental and physical health outcomes via greater sleep disturbance. While prior literature shows associations between stigma and discrimination and mental health and physical health outcomes, few studies have tested potential transdiagnostic mechanisms such as sleep disturbance that are modifiable via low cost interventions, relatively less stigmatized, and could be targets of intervention efforts to reduce health disparities [41–44].

## Methods

### Participants and Procedures

Data collection for this study was conducted between January 2018 and June 2021 in Los Angeles County, California, at a community-based HIV service organization that partnered with the study team. Survey assessments prior to the COVID-19 pandemic (starting March 13, 2020) were conducted in person; during the COVID-19 pandemic, survey assessments were conducted via telephone instead of in person. Participants were Black men and women who participated in a RCT of a culturally congruent behavioral intervention to improve ART adherence, retention in care, and viral suppression in Black adults with HIV [45]. The inclusion criteria for the study were: (1) Black/African American racial/ethnic identity, (2) HIV-positive serostatus, (3) 18 years of age or above, (4) prescribed ART for at least 6 months, (5) self-reported adherence problems (i.e., missed at least one ART dose in the past month) and/or detectable viral load; (6) willing to use an electronic adherence monitoring device; and (7) able to communicate in English. The current analysis included participants from both intervention and control groups and data collected at baseline, 7-month follow-up, and 13-month follow-up. Intervention condition was controlled for in all analyses. There were 245 participants in the RCT; the current analyses included 193 participants (after restricting the sample to participants who responded to the baseline and at least one of the two follow-up surveys).

The institutional review board at the RAND corporation approved the study procedures. All participants provided informed consent and received $30 for completing the baseline assessment, $40 for the 7-month follow-up, and $50 for the 13-month follow-up. The research team partnered with the Community Advisory Board (CAB) at the community-based organization partner APLA Health throughout the study period to elicit input on study design, recruitment, and result interpretation. CAB members included clients and staff members from the partner organization and other community-based organizations primarily serving Black people with HIV.

### Measures

#### Socio-demographic Characteristics

A self-report questionnaire was used to obtain information about the participant's age, sex at birth, gender identification (cisgender man, cisgender woman, transgender or genderqueer/non-conforming), sexual orientation, Latinx ethnicity, education attainment, current employment status, housing status in the past year, annual household income, marital status, incarceration history, and number of years since HIV diagnosis. Table 1 shows these socio-demographic characteristics at baseline.

**Table 1:** Sample characteristics at baseline (*N* = 193)

Variables	Mean (*SD*) or %
Age	49.0 (12.1)
Female at birth	38 (19.4%)
Gender identity	
Cisgender male	145 (75.4%)
Cisgender female	38 (19.4%)
Transgender or genderqueer/non-conforming	10 (15.2%)
Sexual orientation	
Straight or heterosexual	51 (26.6%)
Gay or homosexual man or same gender loving	112 (58.1%)
Lesbian or homosexual woman	2 (1.1%)
Bisexual	25 (12.8%)
Something else	3 (1.4%)
Latinx Ethnicity	17 (9.1%)
Educational attainment	
1st through 11th grade	25 (12.8%)
High school diploma or GED	58 (29.7%)
Some college, but no degree	66 (33.7%)
College degree	31 (16.6%)
Some graduate coursework, but no degree	10 (5.6%)
Graduate degree	3 (1.6%)
Employment status	
Employed, working 40 or more hours per week	8 (4.8%)
Employed, working 1–39 h per week	18 (10.5%)
Not employed, looking for work	31 (15.9%)
Not employed, not looking for work	11 (5.8%)
Retired	23 (11.0%)
Disabled, not able to work	96 (48.8%)
Student	6 (3.1%)
No unstable housing in the past year	104 (52.6%)
Annual household income	
I currently have no income	22 (11.7%)
More than $0 but less than $10,000	64 (32.3%)
$10,000-$20,000	84 (44.8%)
$20,001-$30,000	14 (7.6%)
$30,001-$40,000	3 (1.9%)
Over $40,000	3 (1.8%)
Marital status	
Single	152 (79.9%)
Married/ living with significant other	19 (9.3%)
Divorced / separated /widowed	21 (10.7%)
Ever been incarcerated since age 18	94 (50.3%)
Years since HIV diagnosis	17.1 (9.6)

All means, *SD*s, and percentages are weighted to account for response to at least one of the two follow-up surveys, while *N*s are not weighted. Table includes baseline data for the 193 participants who responded to at least one of the two follow-up surveys

#### Internalized HIV Stigma

The Internalized AIDS-Related Stigma Scale was used to assess internalized HIV stigma [46]. Six items were rated on a 5-point Likert scale (1 = strongly disagree, 2 = slightly disagree, 3 = not sure or have no opinion, 4 = slightly agree, 5 = strongly agree). Sample items include: “it is difficult to tell other people about my HIV infection,” “being HIV positive makes me feel dirty,” and “I hide my HIV status from others.” The reliability for this measure was high at baseline (*⍺* = 0.88).

#### Anticipated HIV Stigma

Anticipated HIV stigma was assessed using four items assessing anticipated HIV stigma among family and friends and four items assessing anticipated HIV stigma among providers [9, 10]. Sample items include: “A friend or family will blame you for not getting better,” “a friend or family will think that your illness is your fault,” “a healthcare worker will be frustrated with you,” and “a healthcare worker will think that you are a bad patient.” The reliability coefficients were high for both set of items (*⍺* = 0.90 for family/friends, *⍺* = 0.89 for healthcare workers) and for all eight items (*⍺* = 0.87). Therefore, we used the summary score for all eight items in the analysis.

#### Perceived Discrimination

The Multiple Discrimination Scale was used to measure experiences with ten different discrimination events in the past year (at baseline) and past 6-months (at both 7-month and 13-month follow-up) related to being HIV-positive, being Black, and being perceived as gay [11]. Sample items from this measure include: "In the past year/6-months, were you ignored, excluded, or avoided by people close to you because [you are HIV positive / you are Black or African American/ someone thought you were gay]?" "In the past year/6-months, were you denied a place to live or did you lose a place to live because [you are HIV positive / you are Black or African American / someone thought you were gay]?", and "In the past year/6-months, were you physically assaulted or beaten up because [you are HIV positive / you are Black or African American / someone thought you were gay]?" The participant experienced each of the ten types of discrimination events (1, yes or 0, no), and we derived summary scores of the binary responses to the ten items (possible range: 0–10) for each subscale/identity. To address the differences in timeframes and, therefore, means of the summary scores, we divided the baseline subscale scores (asked for *past year*) by two so that the baseline means were consistent with the 7-month and 13-month follow-up (asked for *past 6-month*), assuming that the responses would be proportional to the timeframe. Reliability coefficients for the three subscales in the current study were high at baseline: 0.87 (HIV-positive), 0.91 (Black), and 0.92 (gay).

#### Sleep Disturbance

Sleep disturbance was measured using one item (item 3) from the Patient Health Questionnaire depression scale (PHQ-8) “Over the *last 2 weeks*, how often have you been bothered by having trouble falling or staying asleep, or sleeping too much?” This item was rated on a 4-point scale (0 = not at all, 1 = several days, 2 = more than half the days, 3 = nearly everyday). Prior evidence supports the use of this item as a screening test for sleep problems as it shows a significant correlation with scores of sleep scales such as the Insomnia Severity Index [47–49].

#### Mental Health

Depressive symptoms were measured using the eight-item Patient Health Questionnaire depression scale (PHQ-8) [50]. Each item was rated on a 4-point scale: 0 = not at all, 1 = several days, 2 = more than half the days, 3 = nearly everyday. Following prior research examining sleep and depression as measured by the PHQ [51, 52], the sleep item was removed from the summary score used in the current analysis. A summary score was derived by summing all seven items (excluding the sleep item), resulting in 0–21 possible range.

PTSD symptom count was measured using the primary care PTSD screen (PC-PTSD) [53]. PC-PTSD assesses four PTSD symptoms about any experience that was frightening, horrible, or upsetting: “have had nightmares about it,” “tried hard not to think about it,” “were constantly on guard, watchful, or easily startled” “felt number or detached from others, activities, or surroundings.” The participant was asked to rate whether they have experienced each symptom (1 = Yes; 0 = No) in the past month. The current analysis used the sum of the symptom count, with a possible range between 0 and 4.

#### Health-Related Quality of Life (HRQOL)

Health-Related Quality of Life was measured using eight items from the Patient-Reported Outcomes Measurement Information System (PROMIS) [54]. Four items were used to measure global physical health, including items on overall physical health, physical function, pain, and fatigue; four items were used to measure global mental health, including items on quality of life, mental health, satisfaction with social activities, and emotional problems. Each item was rated on a 5-point Likert scale (1 = Poor, 2 = Fair, 3 = Good, 4 = Very good, 5 = Excellent). Continuous summary scores were derived for physical and mental health, respectively.

### Data Analysis

Multiple linear regression analyses were conducted using SAS v9.4 (©2016 SAS Institute Inc.). The analytic sample included 193 participants who completed the baseline survey and at least 1 (7-month or 13-month) follow-up survey. Of 245 participants randomized at baseline, 70 participants were missing 7-month follow-up and 79 were missing 13-month follow-up; 52 were missing both follow-ups and excluded from this analysis. To reduce unit nonresponse bias, all analyses were adjusted with nonresponse weights. Nonresponse weights were derived as the inverse of the predicted probability of completing at least one follow-up survey, modeled via logistic regression with thirteen sociodemographics measured at baseline. To assess the impact of stigma and discrimination on sleep and health outcomes, we performed linear regression models using PROC SURVEYREG, with adjustments for nonresponse weighting to account for nonresponse bias as well as clustering on individual ID to account for repeated measures of each outcome across the three time points.

To estimate the indirect effects of HIV stigma and discrimination measures on mental and physical health outcomes via sleep disturbance, we used Stata 18 to conduct causal mediation analysis. For each health outcome, we conducted two sets of linear models accounting for repeated measures across three waves. The first estimated the association between HIV stigma or discrimination and sleep disturbance. The second examined the relationship between sleep disturbance and the health outcome, adjusting for HIV stigma or discrimination. Each model accounted for age, sex assigned at birth, and intervention condition. We initiated causal mediation analysis by predicting the mediator across contrasting levels of the predictor, specifically comparing internalized or anticipated HIV stigma between -1 and + 1 *SD* (or from the 25th to the 75th percentiles for discrimination measures). The analysis proceeded to predict the health outcome with and without the mediator, taking the difference to estimate the mediated effect. We then used bootstrapping (with 2000 replications) to assess uncertainty [55]. The direct and indirect effects were adjusted for the clustering of repeated measures. The bootstrapped standard errors and a 95% confidence interval excluding zero were used to determine statistical significance at *p* < 0.05 [56].

Age, sex assigned at birth, and treatment condition were included as covariates in all adjusted models. In addition, we evaluated a list of potential covariates and included them only if they had a statistically significant (*p* < 0.05) bivariate association with both the outcome and the predictor variable in each model. The list of covariates evaluated included sexual orientation (coded as heterosexual versus gay, lesbian, bisexual or something else), employment status (coded as employed versus not), marital status (coded as married versus not), incarceration history (yes/no), stable housing in the past year (yes/no), Hispanic ethnicity, education level (coded as 11th grade or lower versus high school or greater), income (coded as < $10,000 versus $10,000 or greater annually), and years since HIV diagnosis. In the final models, we included covariates that were associated with both the predictor and the outcome variables. Specifically, incarceration history was added for models where the dependent variable was PTSD and predictor variable was anticipated stigma or HIV discrimination; employment status was added for the model where the dependent variable was HRQ physical health and the predictor variable was Black discrimination.

## Results

Table 1 shows the sample characteristics at baseline for the analytic sample (*N* = 193). Participants were, on average, 49.0 years of age and had been living with HIV for about 17.1 years. About one fifth (19.4%) were assigned female at birth, and most of the sample (72.0%) self-identified as gay or bisexual. Most participants (87.2%) held at least high school diploma, while only 15.3% were employed either full-time or part-time, and 44.0% had an annual household income below $10,000. About half (52.6%) did not experience unstable housing in the past year, about half had ever been incarcerated as adults (50.3%), and most (79.9%) were single. Table 2 summarizes the descriptive statistics of all study variables across time points. The Supplemental Table shows the correlation between sociodemographic variables and study variables at baseline.

**Table 2:** Descriptive statistics for all study variables at baseline, 7-month, and 13-month follow-up

Variables	Baseline			7-month follow-up			13-month follow-up		
	Mean	SD	Range	Mean	SD	Range	Mean	SD	Range
Internalized HIV stigma	2.82	1.25	1 – 5	2.54	1.14	1 – 5	2.38	1.11	1 – 5
Anticipated HIV stigma	2.04	0.81	1 – 4.5	1.96	0.79	1 – 5	1.92	0.78	1 – 4.75
Multiple discrimination -HIV	1.28	2.24	0 – 10	0.77	1.72	0 – 10	0.62	1.50	0 – 9
Multiple discrimination—Black	1.98	2.84	0 – 10	1.02	1.98	0 – 10	1.10	1.91	0 – 9
Multiple discrimination -Gay	1.48	2.61	0 – 10	0.88	1.90	0 – 10	0.83	1.59	0 – 9
Sleep problem	1.08	1.03	0 – 3	1.02	1.01	0 – 3	0.85	0.94	0 – 3
PHQ* (without sleep item)	5.92	5.49	0 – 21	5.32	4.90	0 – 21	4.91	4.79	0 – 21
Number of PTSD symptoms	1.33	1.58	0 – 4	1.10	1.32	0 – 4	1.20	1.31	0 – 4
HRQOL mental health scale	2.79	0.86	1 – 5	3.01	0.84	1 – 5	3.02	0.81	1 – 5
HRQOL physical health scale	3.21	0.81	1.25 – 5	3.45	0.75	1.75 – 5	3.45	0.79	1.25 – 5

*HRQOL* Health-Related Quality of Life, PHQ* Patient Health Questionnaire depression scale (PHQ-8), without item 3 (sleep problem), *PTSD* PC-PTSD total score. The means for the multiple discrimination subscales at baseline were the original, uncorrected means. All means and *SD*s are weighted to account for response to at least one of the two follow-up surveys. Note that no significant baseline differences were found in nine out of the ten study variables (shown in this table) between those who completed follow-ups and those lost to follow-up; however, there was a significant difference in HIV-related discrimination, with those lost to follow-up reporting higher levels at baseline (*M* = 1.93, *SD* = 2.51) compared to those who completed follow-ups (*M* = 1.23, *SD* = 2.20, *p* = .01)

Table 3 summarizes the results from linear models examining the impact of HIV stigma (internalized and anticipated) and multiple discrimination (related to HIV-serostatus, Black race, and being perceived as gay) on sleep, depressive symptoms (excluding the item that assesses sleep disturbance), PTSD symptoms, HRQOL mental health, and HRQOL physical health. Estimates were consistent comparing across unadjusted and adjusted models; therefore, only adjusted estimates that were statistically significant (*p* < 0.01) are presented in Table 3. Specifically, across three measurements (at baseline, 7-month, and 13-month follow-up), greater levels of internalized and anticipated HIV stigma as well as HIV-serostatus, Black race, and sexual orientation discrimination predicted greater sleep disturbance, higher depressive symptoms as indexed by PHQ (without the sleep item), more PTSD symptoms, and worse HRQOL in both mental and physical health domains.

**Table 3:** Results from mixed effects model examining the impact of stigma and discrimination on sleep, mental health, and physical health outcomes measured at baseline, 7-month, and 13-month follow-up

	Sleep disturbance	PHQ*	PTSD	HRQOL mental health	HRQOL physical health
Predictors	Estimate (SE)	Estimate (SE)	Estimate (SE)	Estimate (SE)	Estimate (SE)
Internalized HIV stigma	0.22 (0.05) ***	1.59 (0.24) ***	0.38 (0.07) ***	-0.26 (0.04) ***	-0.14 (0.04) **
Anticipated HIV stigma	0.30 (0.07) ***	2.24 (0.38) ***	0.55 (0.10) ***	-0.37 (0.05) ***	-0.28 (0.05) ***
Multiple discrimination—HIV	0.21 (0.04) ***	1.40 (0.25) ***	0.41 (0.05) ***	-0.18 (0.03) ***	-0.14 (0.02) ***
Multiple discrimination—Black	0.14 (0.03) ***	0.99 (0.19) ***	0.33 (0.04) ***	-0.11 (0.03) ***	-0.09 (0.03) **
Multiple discrimination—Gay	0.14 (0.03) ***	0.92 (0.23) ***	0.28 (0.05) ***	-0.12 (0.02) ***	-0.10 (0.03) ***

*HRQOL* Health-Related Quality of Life, ^*^PHQ Patient Health Questionnaire depression scale (PHQ-8), without item 3 (sleep problem). *** indicates *p* < 0.001; ** indicates *p* < .01. The estimates are unstandardized regression coefficients. All models controlled for age, sex at birth, and intervention condition, as well as additional covariates that were significant for specific combinations of dependent variable and predictor (incarceration history for the models where outcome was PTSD and predictor variable was anticipated stigma or HIV discrimination; and employment status for the model where outcome was HRQ-physical health and predictor variable was Black discrimination)

Table 4 summarizes the estimates of the indirect effects of HIV stigma and discrimination on health outcomes via sleep disturbance. All bootstrap 95% CI of the indirect effect estimates did not include zero, indicating significant indirect effects via sleep disturbance. For example, greater internalized HIV stigma was associated with higher depressive symptoms and PTSD symptoms, and there were significant indirect effects via higher sleep disturbance (indirect effect: 1.71, bootstrap 95% CI [0.88, 2.55] for depressive symptoms; 0.29, bootstrap 95% CI [0.15, 0.44] for PTSD symptoms). Greater internalized HIV stigma was associated with worse HRQOL in both mental and physical health domains. There were significant indirect effects via higher sleep disturbance: indirect effect = -0.17, bootstrap 95% CI [-0.25, -0.08] for HRQOL mental health; indirect effect = -0.08, 95% CI [-0.18, -0.05] for HRQOL physical health. The indirect effects were in the same direction between other predictors and outcomes.

**Table 4:** Estimates of the indirect effects via sleep problems

Predictor	Outcome	Indirect Effects	Bootstrap SE	Bootstrap 95% CI	% mediated [Bootstrap 95% CI]
Internalized HIV stigma	PHQ*	**1.71**	**0.43**	**[0.88, 2.55]**	**45% [33%, 52%]**
	PTSD symptoms	**0.29**	**0.08**	**[0.15, 0.44]**	**33% [25%, 38%]**
	HRQOL mental health	**-0.17**	**0.04**	**[-0.25, -0.08]**	**26% [31%, 17%]**
	HRQOL physical health	**-0.12**	**0.03**	**[-0.18, -0.05]**	**34% [36%, 30%]**
HIV anticipated stigma	PHQ*	**1.58**	**0.40**	**[0.81, 2.36]**	**43% [32%, 49%]**
	PTSD symptoms	**0.25**	**0.07**	**[0.11, 0.39]**	**26% [17%, 31%]**
	HRQOL mental health	**-0.15**	**0.04**	**[-0.23, -0.07]**	**24% [28%, 16%]**
	HRQOL physical health	**-0.11**	**0.03**	**[-0.18, -0.05]**	**23% [27%, 15%]**
Multiple discrimination -HIV	PHQ*	**0.45**	**0.09**	**[0.29, 0.62]**	**49% [49%, 50%]**
	PTSD symptoms	**0.07**	**0.02**	**[0.04, 0.10]**	**19% [14%, 22%]**
	HRQOL mental health	**-0.05**	**0.01**	**[-0.07, -0.03]**	**35% [36%, 32%]**
	HRQOL physical health	**-0.04**	**0.01**	**[-0.06, -0.02]**	**31% [33%, 27%]**
Multiple discrimination—Black	PHQ*	**0.64**	**0.14**	**[0.37, 0.91]**	**50% [43%, 53%]**
	PTSD symptoms	**0.10**	**0.02**	**[0.05, 0.14]**	**20% [14%, 24%]**
	HRQOL mental health	**-0.07**	**0.02**	**[-0.10, -0.04]**	**41% [40%, 44%]**
	HRQOL physical health	**-0.05**	**0.01**	**[-0.08, -0.03]**	**36% [37%, 34%]**
Multiple discrimination -Gay	PHQ*	**0.27**	**0.07**	**[0.13, 0.42]**	**51% [44%, 53%]**
	PTSD symptoms	**0.05**	**0.01**	**[0.02, 0.07]**	**24% [16%, 28%]**
	HRQOL mental health	**-0.03**	**0.01**	**[-0.05, -0.01]**	**35% [38%, 27%]**
	HRQOL physical health	**-0.02**	**0.01**	**[-0.04, -0.01]**	**32% [35%, 26%]**

PHQ* Patient Health Questionnaire depression scale, without item 3 (sleep problem). Sleep problem was measured using PHQ item 3 (how often bothered with sleep in the past 2 weeks). Boldface indicates significant indirect effects. All indirect effect estimates were significant (none of the bootstrap 95% CIs included zero). % mediated was calculated using estimates of the indirect effects divided by total effects (not reported in the table)

## Discussion

This study used longitudinal data from a recent RCT to examine associations between several dimensions of HIV-related stigma and discrimination related to multiple identities and sleep disturbance, mental health, and physical health in a sample of Black adults with HIV. In addition, sleep was tested as an indirect/mediating path for the impact of stigma and discrimination on mental and physical health. Consistent with our hypotheses, we found that internalized HIV stigma, anticipated HIV stigma, and perceived discrimination (enacted stigma) related to being HIV positive, Black, and perceived as gay were associated with greater sleep problems, depressive symptoms, PTSD symptoms, and poor mental and physical HRQOL across baseline, 7-month follow-up, and 13-month follow-up. In addition, greater sleep disturbance measured at 7-month follow-up mediated the impact of HIV-related stigma and multiple discrimination measured at baseline on worse ratings of mental and physical health (i.e., more depressive and PTSD symptom endorsement, worse mental and physical HRQOL).

Our results are consistent with the larger literature documenting the impact of different forms of stigma on mental and physical health among people with HIV in general [9, 10, 57] and in populations such as Black Americans that are affected most by the HIV disparities [11, 12]. Our results add to evidence that documents the impact of stigma and poor health outcomes in a sample of Black men and women with HIV, who are underrepresented in health research despite showing the largest HIV disparities. Findings are also consistent with the theories and evidence demonstrating that health-related stigmas, including internalized, anticipated, and enacted stigma, can lead to sleep disturbance and deficiencies [30, 31], which, in turn, are a contributory causal factor to mental and physical health outcomes [33, 35]. The current results add to the existing literature by examining enacted stigma from other social identities (i.e., race, sexual orientation) and by showing that sleep disturbance at least in part explains the associations between health-related stigma and discrimination and health outcomes using longitudinal data.

Several limitations should be noted. First, we relied on a single-item, self-reported rating of sleep disturbance from a validated measure in the current study. While there is evidence that a single item could adequately assess the perception of sleep disturbance/quality [58], including in some studies examining similar relationships (e.g., perceived discrimination and sleep) [31], and the sleep item from PHQ has been empirically supported as a screener [47–49], future research should use multi-item measures, incorporate objective assessment of sleep, and assess multiple dimensions of sleep health (including sleep quality, duration, efficiency, regularity, satisfaction, and daytime impairment such as sleepiness). Relatedly, the current study relied on self-reported data for assessing sleep disturbance, stigma experiences, and health outcomes, which can introduce recall and social desirability biases. Future research should incorporate objective measures such as actigraphy for sleep measurement. We also acknowledge the potential limitation of unmeasured variables that could affect the associations between HIV stigma, sleep disturbance, and health outcomes; future studies should include a broader range of covariates (e.g., access to healthcare, number of comorbidities) to enhance understanding of these mechanisms. Additionally, while our longitudinal design enhances the inference of causal relationships, bidirectional influences may still exist where poor health outcomes could exacerbate perceived stigma or sleep disturbances. Furthermore, our multiple discrimination measure used different time frames at baseline and follow-ups; for the purposes of linear regression models, we divided baseline values in half so that means would be consistent over time, assuming that the responses would be proportional to the time frame used. We believe this assumption is reasonable, given that the means of discrimination experiences for the past 6 months were about half of the mean for the past year. Another potential limitation arises from the higher levels of HIV-related discrimination reported by participants lost to follow-up, which suggests our results may underrepresent the full impact of discrimination on health outcomes. Finally, although the data for this analysis were derived from a randomized controlled trial, in which one group received an intervention focused on problem-solving regarding medication adherence, we accounted for intervention condition in our analyses by adding it as a covariate in all models.

In conclusion, in a sample of Black Americans with HIV, we examined the relationship between HIV-related stigma and discrimination based on multiple stigmatized identities and mental and physical health outcomes and tested whether sleep disturbance mediated this relationship. Our results indicate that internalized and anticipated HIV stigma and experiences of multiple discrimination based on being HIV-positive, being Black, and being gay are important contributors to poor mental and physical health in this population. Sleep disturbance may be an indirect path that is modifiable and potentially less stigmatized (relative to HIV and mental disorders) [44] to target in interventions. Intervention efforts should reduce sources of intersectional stigma and discrimination. For example, we examined anticipated HIV stigma by family, friends, and healthcare providers, who could be a target of the intervention to reduce perceived stigma and discrimination. To alleviate the impact of intersectional stigma and discrimination at the individual level, interventions could target underlying processes such as sleep disturbance, which may result in broader benefits across mental and physical health domains. Future research on interventions should explore barriers to sleep disturbance in this population (e.g., resource constraints, accessibility of services) and adapt strategies to improve sleep, examining their impact on other health domains.

## Supplementary Information

Below is the link to the electronic supplementary material.

Supplementary file1 (DOCX 17 KB)

## Data Availability

De-identified data can be requested after completing a Data User Agreement.

## Code Availability

Data analysis codes are available upon request.
