# Supplementary material for: Sleep Disturbance Mediates the Associations Between HIV Stigma and Mental and Physical Health Among Black Adults with HIV
Source: J Racial Ethn Health Disparities. 2024 Jul 11;12(4):2707–16. doi: 10.1007/s40615-024-02083-0 (PMC11724012; doi:10.1007/s40615-024-02083-0)
Supplement: Supplementary file 1 — Supplementary file1 (DOCX 17 KB) [file 40615_2024_2083_MOESM1_ESM.docx]

**Supplemental Table**

*Correlation of Sociodemographics with all Study Variables at Baseline*

**Pearson correlation coefficient**

| **Variables** | Age | Female | Hetero-sexual | Latinx | Edu-cation | Working | Stable housing | Annual HH income | Married | Ever jailed since age 18 | Years since HIV diagnosis |
| --- | --- | --- | --- | --- | --- | --- | --- | --- | --- | --- | --- |
| Internalized HIV stigma | **-0.19** | 0.04 | 0.11 | -0.03 | 0.12 | 0.04 | -0.10 | -0.04 | -0.03 | 0.11 | **-0.25** |
| Anticipated HIV stigma | -0.09 | 0.11 | 0.11 | 0.03 | 0.07 | -0.08 | -0.10 | -0.03 | **-0.15** | **0.17** | -0.10 |
|  |  |  |  |  |  |  |  |  |  |  |  |
| Multiple discrimination -HIV | **-0.22** | -0.03 | 0.01 | 0.06 | **0.19** | 0.13 | -0.03 | 0.08 | -0.12 | **0.17** | **-0.27** |
| Multiple discrimination - Black | **-0.15** | -0.04 | 0.02 | 0.06 | **0.30** | **0.19** | -0.09 | 0.12 | -0.07 | 0.08 | **-0.16** |
| Multiple discrimination -Gay | **-0.24** | **-0.15** | -0.09 | 0.14 | **0.16** | 0.12 | -0.06 | 0.06 | **-0.17** | 0.13 | **-0.23** |
|  |  |  |  |  |  |  |  |  |  |  |  |
| Sleep problem | -0.02 | -0.02 | 0.10 | 0.03 | 0.00 | 0.03 | -0.05 | 0.05 | -0.05 | 0.00 | -0.05 |
| PHQ* (without sleep item) | **-0.21** | 0.05 | 0.03 | 0.06 | 0.11 | 0.10 | -0.09 | -0.01 | -0.10 | 0.07 | **-0.21** |
| Number of PTSD symptoms | -0.10 | -0.06 | -0.01 | 0.11 | 0.08 | 0.04 | -0.12 | 0.01 | -0.06 | **0.18** | -0.07 |
| HRQOL mental health scale | 0.09 | -0.02 | 0.01 | -0.07 | -0.11 | -0.08 | **0.15** | 0.03 | 0.14 | -0.06 | **0.17** |
| HRQOL physical health scale | **-0.20** | **-0.14** | **-0.19** | -0.04 | -0.01 | **0.23** | 0.11 | 0.04 | 0.07 | -0.05 | -0.09 |

*Note*. PHQ* =Patient Health Questionnaire depression scale (PHQ-8), without item 3 (sleep problem). PTSD = PC-PTSD total score. HRQOL = Health-Related Quality of Life

**Bold** coefficients are statistically significant at p<.05.
